# Transcriptome Analysis Reveals Host Peripheral Blood Mononuclear Cells Response to Mpox Virus Infection

**DOI:** 10.3390/v17101317

**Published:** 2025-09-28

**Authors:** Chaode Gu, Caiyun Wang, Chenlu Zhang, Jie Ni, Yun Xia, Hongwei Wang

**Affiliations:** 1 State Key Laboratory of Analytical Chemistry for Life Science & Jiangsu Key Laboratory of Molecular Medicine, Medical School, Nanjing University, Nanjing 210093, China; chaode_gu@smail.nju.edu.cn (C.G.); m18551893985@163.com (C.W.); zhang2023chenlu@163.com (C.Z.); 2 Department of Respiratory Medicine, Children’s Hospital of Nanjing Medical University, Nanjing 210008, China; 3 Jinling Pharmaceutical Company Limited, Jinling Pharmaceutical Building, No. 238 Zhongyang Road, Nanjing 210009, China; 4 Suqian Scientific Research Institute of Nanjing University Medical School, Nanjing 210008, China

**Keywords:** mpox virus, PBMCs, rabbit, infection

## Abstract

Mpox virus (MPXV), a member of the *Orthopoxvirus* genus in the Poxviridae family, has long been endemic in Africa. The interaction between MPXV infection and peripheral immune responses is of great significance. However, the activation of signaling pathways and molecular changes in peripheral blood mononuclear cells (PBMCs) following MPXV infection remain poorly understood. This study evaluated the transcriptomic profiles of rabbit PBMCs during the mpox acute and recovery phases. The results showed that MPXV infection significantly altered the transcriptomic profiles of PBMCs. At 6 days post-infection, pathways related to pathogenic infection and IL-1 response were enriched, while at 14 days post-infection, the T cell receptor signaling pathway was enriched. During the mpox acute phase, inflammatory cytokines in serum such as IL-1α, IL-1β, IL-8, and IL-21 were upregulated, while MMP-9 and NCAM-1 were downregulated. In rabbits and rhesus monkeys, key genes upregulated in common during the mpox acute period were associated with the interferon pathway (e.g., the *ISG15*, *OAS*, and *IFIT* families), while downregulated genes were related to B-cell activation and differentiation (e.g., the *MS4A1* and *FCRL* families). Additionally, rabbits developed protective immunity against reinfection, with neutralizing antibodies effectively activated. These findings provide insights into the molecular characteristics of PBMCs changes in in vivo models of MPXV infection, and offer references for the diagnosis, vaccine development, and therapeutic research of mpox.

## 1. Introduction

As a member of the genus *Orthopoxvirus* in the family Poxviridae, mpox virus (MPXV) has long been endemic in Africa. In 2022, MPXV triggered a cross-species transmission epidemic across multiple non-endemic countries worldwide. Data from the World Health Organization (WHO) showed that this mpox outbreak resulted in over 99,000 confirmed cases across more than 118 countries [1], challenging the traditional perception of “limited human-to-human transmission capacity”. Although the global mpox epidemic has been effectively controlled, the situation remains grim in Africa [2,3], posing a long-term potential threat to the global public health system [4,5,6].

The progression of MPXV infection is closely associated with peripheral immune responses. In non-human primate (NHP) models of MPXV infection, the proportions of granulocytes and monocytes are increased [7]. In lethal MPXV infection models, monocytes and granulocytes are the primary cell types positive for poxvirus antigens [8]. MPXV can promote serum Th2 responses, as evidenced by elevated levels of cytokines such as IL-4, IL-5, and IL-6 [9]. Concentrations of GM-CSF, IL-10, and sIL-2R are significantly elevated in serum samples from severe cases [9]. MPXV promotes the production of IFN-β by activating the cGAS-STING pathway, and IFN-I can reduce the pathogenicity of MPXV challenge in mice and rhesus macaques [10]. Immunocompromised individuals, such as patients with advanced HIV infection (CD4 count < 350 cells/μL), experience more severe disease symptoms or even death following MPXV infection [11]. These studies have enhanced our understanding of the interplay between MPXV infection and peripheral immune responses. However, the activation of signaling pathways and molecular changes in peripheral blood mononuclear cells (PBMCs) post-MPXV infection remain poorly understood; to date, transcriptomic changes in PBMCs following MPXV infection have only been reported in NHP models [12]. Compared with NHPs, rabbits offer advantages such as lower cost and faster reproduction, while also recapitulating typical MPXV infection symptoms. Nevertheless, research on rabbit PBMCs post-MPXV infection is completely lacking, and there is no data supporting comparisons of molecular response patterns between rabbits and primates.

Herein, we evaluated the transcriptomic profiles of rabbit PBMCs during the mpox acute and recovery phases, identified differentially expressed genes (DEGs), and enriched key signaling pathways. We further validated the expression of critical inflammatory factors using enzyme-linked immunosorbent assay (ELISA). By integrating transcriptomic data of MPXV-infected NHP PBMCs from public databases, we analyzed conserved signature molecules in PBMCs during the mpox acute phase across the two models. Finally, we verified the neutralizing antibody levels in rabbits following MPXV reinfection. This study is the first to report the transcriptomic data of rabbit PBMCs infected with MPXV, validate the feasibility of rabbits as a surrogate model, and identify cross-species conserved molecules, thereby providing novel candidate targets for dissecting MPXV immunological mechanisms and advancing translational research.

## 2. Materials and Methods

### 2.1. Cells and Viruses

Vero-E6 cells were cultured in Dulbecco ‘s modified Eagle ‘s medium (DMEM) (Bio-Channel, Nanjing, China, Cat#BCM005) supplemented with 10% fetal bovine serum (FBS) (Bio-Channel, Cat#BCSEFBS01), 50 IU/mL penicillin and 50 μg/mL streptomycin (Gibco, Miami, FL, USA) at 37 °C in a humidified incubator with 5% CO_2_. The MPXV strain (Genebank: PP778666.1) was provided by the Changchun Veterinary Research Institute. The virus was propagated in Vero E6 cells and stably passaged for 3 times. Finally, MPXV-infected cells were repeatedly frozen and thawed three times, and the supernatant containing the virus was collected by centrifugation at 1000× *g* for 10 min. All the experiments of infectious MPXV were carried out in biosafety level 3 laboratory.

### 2.2. Animals

The eight-month-old rabbits (weighing 2.2–2.8 kg) used in this experiment were purchased from Sipeifu (Beijing, China). Rabbits in the infection group were injected with 10^6^ TCID_50_ MPXV in a volume of 1 mL through the ear vein, and three rabbits were injected with virus-free DMEM solution as the Mock group. The body temperature and body weight were measured at 0, 2, 4, 6, 8, 11 and 14 days, and pharyngeal and anal swabs were collected for viral load detection. At 6 and 14 dpi, 3 rabbits of the infected group were sacrificed, and the main organs and PBMCs samples were collected for subsequent detection. Mock was sacrificed at 14 days, and major tissue samples were collected for pathological examination. All animal experiments were approved by the Changchun Veterinary Research Institute Animal Care Committee and followed the Guide for the Care and Use of Laboratory Animals published by the Chinese National Institutes of Health. The research protocols were conducted in strict accordance and adherence to relevant policies regarding animal handling as mandated under the guidelines from the institutional animal care committee (#AMMS- 11-2023-007).

### 2.3. Virus DNA Detection

MPXV DNA was assessed by RT-PCR targeting the F3L gene. The standard curve was fitted using a series of 10-fold dilutions of standard plasmids encoding MPXV-F3L. Tissue samples of heart, liver, spleen, lung, kidney, brain, rectum, testes and skin, throat and anal swabs were collected. Viral DNA in the homogenate was isolated with E.Z.N.A. Viral DNA Kit (Omega, Norcross, GA, USA, Cat#D3892) according to manufacturer ‘s instructions. Viral DNA copy numbers of MPXV in the samples was quantified by RT-PCR using GoTaq qPCR premix (Promega, Madison, WI, USA, Cat#A600A). The primers sequence of MPXV-F3L were as follows: forward: 5′-CAT CTA TTA TAG CAT CAG CAT CAG CAT CAG A-3′ and reverse: 5′-GAT ACT CCT CCT CGT TGG TCT AC-3′. The amplification reaction was performed using the ABI7500 system (Applied Biosystems, Foster, CA, USA) at 95 °C for 2 min, followed by 40 cycles of 95 °C for 15 s and 60 °C for 1 min.

### 2.4. Neutralizing Antibody Detection

The plaque reduction neutralization assay (PRNT_50_) was performed to detect neutralizing antibodies in the serum samples. Vero E6 cells were seeded in 24-well plates at a density of 1 × 10^5^ cells per well and cultured to 90% confluence for subsequent experiments. The inactivated sera (56 °C for 30 min) were diluted 20-fold, followed by a 4-fold serial dilution in DMEM supplemented with 2% rabbit complement serum (vhbio, Cat# CL3411), 50 IU/mL penicillin and 50 μg/mL streptomycin. Each serum sample was incubated with 100 plaque-forming units (PFU) of virus at 37 °C for 1 h. The virus-serum mixture was added to the pre-formed Vero E6 cell monolayer and incubated at 37 °C in a 5% CO_2_ incubator for 1 h. Then, the supernatant was removed and the cell monolayer was covered with methylcellulose (DMEM supplemented with 0.9% methylcellulose, 2% FBS, 50 IU/mL penicillin and 50 μg/mL streptomycin). The neutralizing antibody titers were defined as the serum dilution that resulted in a 50% reduction relative to the total number of plaques counts without antibodies (PRNT_50_). When no neutralization was observed, a value of 10 was assigned.

### 2.5. Transcriptome Sequencing

RNA was extracted and purified by Freezol reagent (Vazyme, Nanjing, China, Cat# R71102). NanoDrop ND-2000 (Thermo, Pittsburgh, PA, USA) was used to evaluate the quality and purity of each sample. Subsequently, the purified RNA samples were submitted to LC Bio Technology (Hangzhou, China) for library preparation and sequencing on the Illumina Novaseq TM 6000 platform (Illumina, San Diego, CA, USA). StringTie 2.2.3 and Ballgown 2.36.0 software were used to estimate the expression level of all transcripts, and the mRNA expression level was calculated based on the number of transcripts per kilobase fragment (-FPKM) per million mapped reads. R package DESeq2 v1.48.1 was used to identify differentially expressed genes (DEGs) with fold changes of >2 or <−2, and *p* < 0.05. Then, gene ontology (GO) enrichment analysis and Kyoto Encyclopedia of Genes and Genomes (KEGG) pathway analysis were performed on the differentially expressed genes in the two groups.

### 2.6. Cytokine Measurement

According to the instructions, cytokines in serum samples were detected by cytokine detection kit (Raybiotech, Guangzhou, China, Cat# QAL-CYT-1), and compared with the standard curve established by the corresponding cytokine standard, and the concentration of each cytokine was calculated.

### 2.7. Statistical Analysis

All statistical analyses were performed using GraphPad Prism 9 software (GraphPad Software, Boston, MA, USA). When *p* < 0.05, the difference was considered statistically significant.

## 3. Results

### 3.1. Symptoms of Rabbits After MPXV Infection

**Figure 1:**
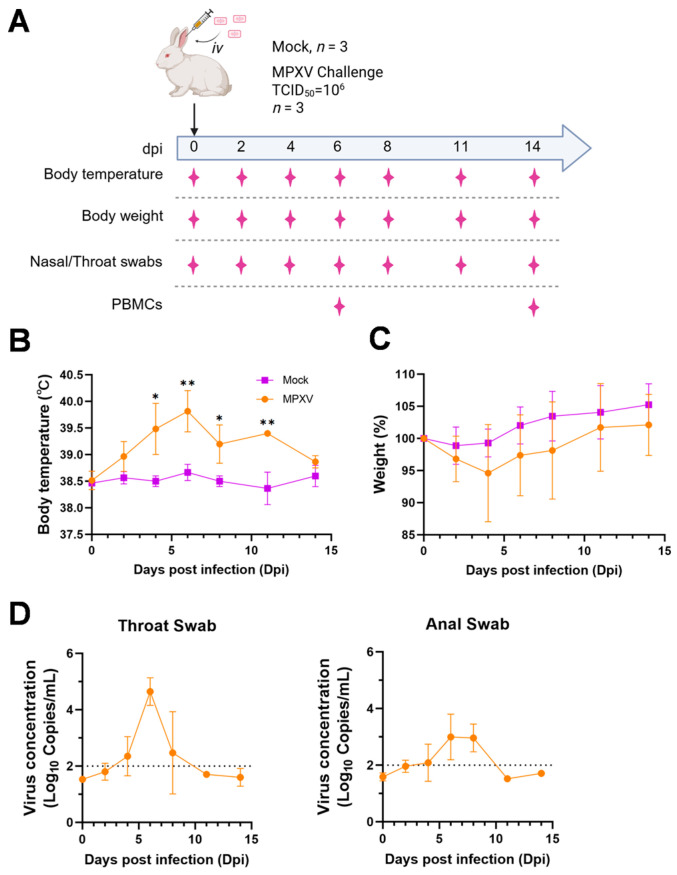
Symptoms of rabbits intravenously injected with MPXV. (**A**) Schematic diagram of MPXV infection and sampling. (**B**,**C**) Body temperature (**B**) and weight changes (**C**) of rabbits in the control group and the infected group. (**D**) The viral copy numbers in the throat and anal swabs of rabbits in the infected group during the course of MPXV infection. Two-way ANOVA was used to determine data significance for weight and temperature. All data were represented as mean ± SEM. * *p* < 0.05, ** *p* < 0.01.

In the present investigation, adult male rabbits were inoculated with 10^6^ TCID_50_ of MPXV via the marginal ear vein (Figure 1A). All inoculated rabbits developed pyrexia, with their body temperatures peaking at 6 days post-infection (dpi), with a mean temperature of 39.8 °C (Figure 1B). A slight decrease in body weight was observed in the infected rabbits, though no statistically significant difference was noted (Figure 1C). Viral DNA was consistently detected in throat swabs from 4 to 8 dpi, reaching the peak at 6 dpi (Figure 1D). Additionally, viral DNA was also detected in rectal swabs from 6 dpi to 8 dpi (Figure 1D), but the peak level was lower than that detected in throat swabs.

### 3.2. Transcriptional Profile of PBMCs in Rabbits Infected with MPXV

**Figure 2:**
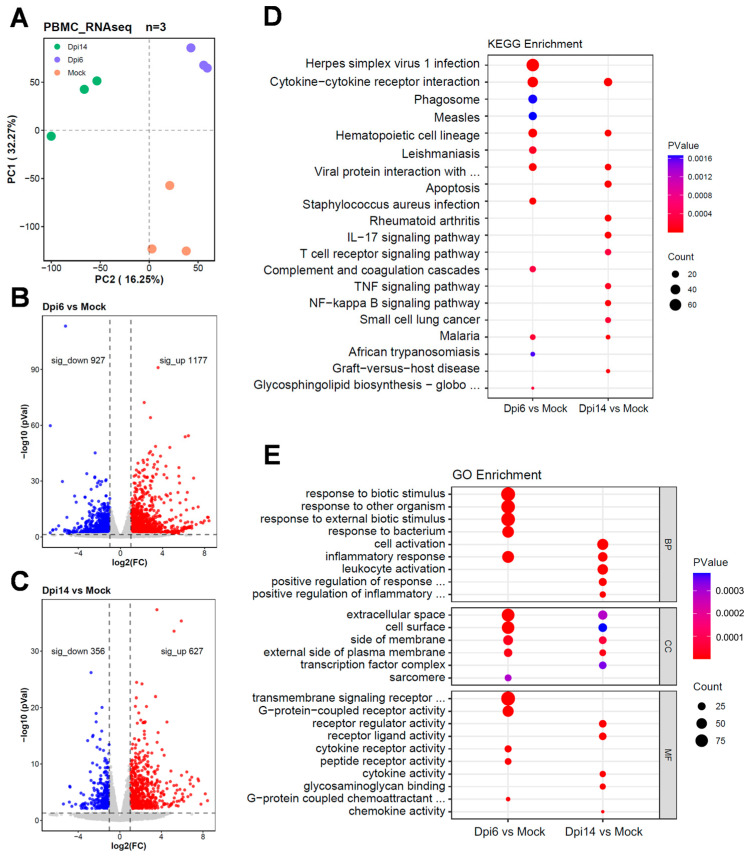
Transcriptomic Analysis of PBMCs in Rabbits Infected with MPXV. Transcriptomic analysis was conducted on the PBMCs of rabbits at 0, 6 and 14 dpi. (**A**) Principal component analysis (PCA). (**B**,**C**) Volcano plots of DEGs in PBMCs at 6 dpi (**B**) and 14 dpi (**C**) after MPXV infection compared to the normal group. (**D**) KEGG pathway analysis of DEGs. The top 12 pathways were shown. (**E**) DEGs were analyzed according to gene ontology terms related to their biological processes (BP), cellular components (CC), and molecular functions (MF). The top 5 pathways in each category were shown.

To describe the inflammatory status and immune milieu of rabbits after MPXV infection, we performed transcriptome sequencing on rabbit PBMCs at 0, 6 and 14 dpi. Results showed that MPXV infection significantly altered the transcriptional levels of genes in PBMCs (Figure 2A). 2104 differentially expressed genes (DEGs) were identified at 6 dpi (Figure 2B), while the number of DEGs decreased to 983 at 14 dpi (Figure 2C). MPXV transcripts were barely detected, with an average reads mapping rate being 7.08 × 10^−6^. This result may be attributed to the limited ability of MPXV to infect PBMCs in in vivo model. At 6 dpi, KEGG pathway enrichment analysis of DEGs revealed that the enriched terms were primarily associated with pathogenic infection (Figure 2D). At 14 dpi, terms such as “IL-17 signaling pathway” and “T cell receptor signaling pathway” were enriched (Figure 2D). For the biological process category in gene ontology (GO) analysis, the most significantly enriched term among DEGs at 6 dpi was “response to biotic stimulus”, whereas terms related to “cell activation” and “leukocyte activation” were most enriched at 14 dpi (Figure 2E).

### 3.3. Expression of Inflammatory Cytokines in PBMCs

**Figure 3:**
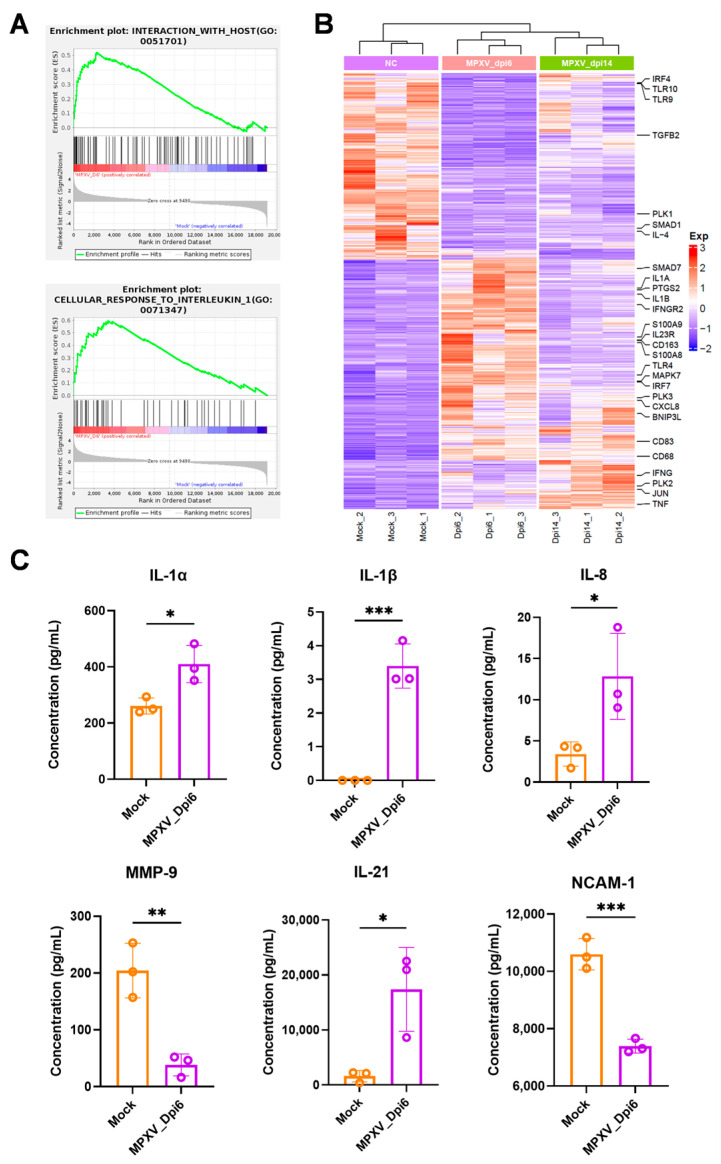
Changes in the inflammatory response of PBMCs from rabbits infected with MPXV. (**A**,**B**) Heatmap of DEGs, which shows the genes related to the inflammatory response. (**C**) Protein levels of IL-1α, IL-1β, IL-8, IL-21, MMP-9, and NCAM-1 in the serum of the normal group and the high-dose infection group at 6 dpi. The significance of cytokine expression levels was determined using the *t*–test. All data were represented as mean ± SEM, * *p* < 0.05, ** *p* < 0.01, *** *p* < 0.001.

We investigated the expression of inflammatory cytokines in PBMCs following MPXV infection. Gene set enrichment analysis (GSEA) revealed that pathways such as “host interaction” and “response to IL-1” were significantly activated at 6 dpi (Figure 3A). The heatmap showed that the transcript levels of genes involved in these pathways (e.g., *IL1A*, *IL1B*, and *CXCL8*) were significantly upregulated at 6 dpi and returned to baseline levels by 14 dpi (Figure 3B). We further evaluated changes in serum inflammatory factors in rabbits using an ELISA chip and confirmed that protein levels of IL-1α, IL-1β, IL-8 and IL-21 were significantly elevated, and MMP-9 and NCAM-1 were downregulated during the mpox acute phase (Figure 3C).

### 3.4. Cross-Species Transcriptomic Analysis of Key Molecular Features During Mpox Outbreaks

**Figure 4:**
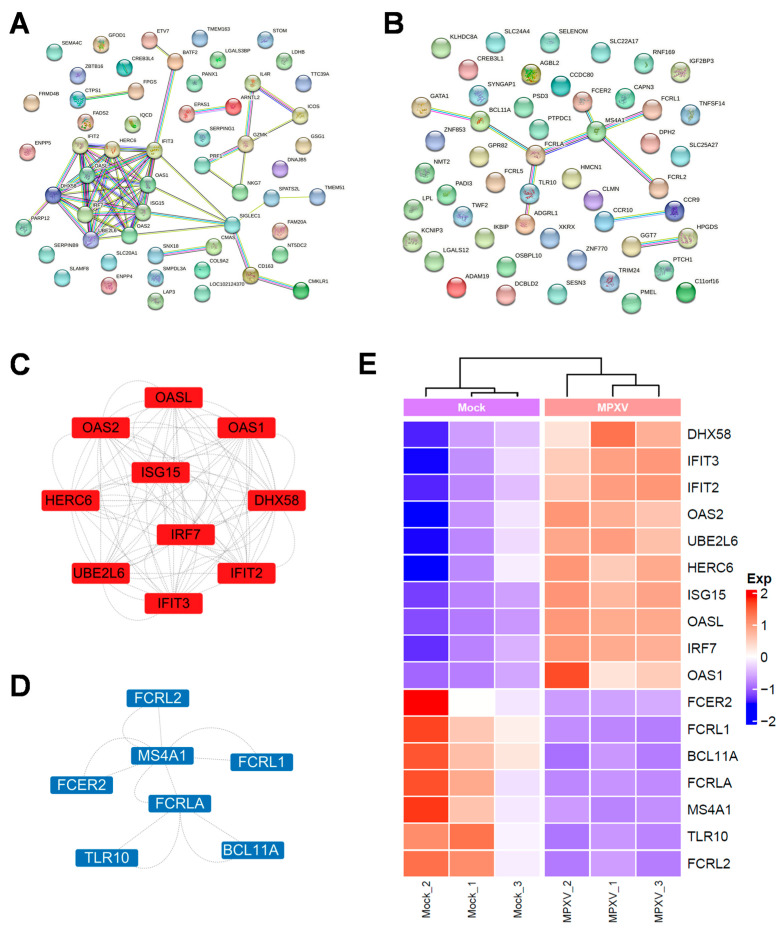
Cross-species transcriptomic analysis of key molecular markers in PBMCs following MPXV infection. (**A**,**B**) Commonly upregulated differentially expressed genes (DEGs) (fold change > 1, *p* < 0.05) and commonly downregulated DEGs (fold change < −1, *p* < 0.05) in rabbits and rhesus macaques during the mpox acute phase. Protein–protein interaction (PPI) networks of these DEGs were constructed using STRING. (**C**,**D**) Key hub genes among the upregulated or downregulated DEGs were analyzed using Cytoscape 3.9.1. (**E**) Heatmap showing the expression profiles of these key hub genes.

To further explore the shared features of PBMCs gene expression patterns between rabbits and other MPXV-infected animal models, we downloaded transcriptomic data of MPXV-infected rhesus macaque PBMCs from a public database (GEO: GSE234118). This dataset corresponds to rhesus macaques infected with 10^6^ TCID_50_ of MPXV clade IIb via intravenous injection, with samples collected at 7 dpi. During the mpox acute phase, PBMCs from the two animal models exhibited 56 upregulated (Figure 4A) and 58 downregulated DEGs in common (Figure 4B). Based on MCODE cluster and GO/KEGG analysis of the PPI network, we further identified hub genes among these DEGs. The key upregulated hub genes were primarily associated with the interferon pathway, such as ISG15, and genes belonging to the OAS and IFIT families (Figure 4C,E). This highlights the critical role of the IFN pathway in the host response to MPXV infection. In contrast, the downregulated hub genes were related to B cells, including *FCRL1* and *FCRLA* (Figure 4D,E).

### 3.5. MPXV-Induced Immunologic Protection in Rabbit Models

**Figure 5:**
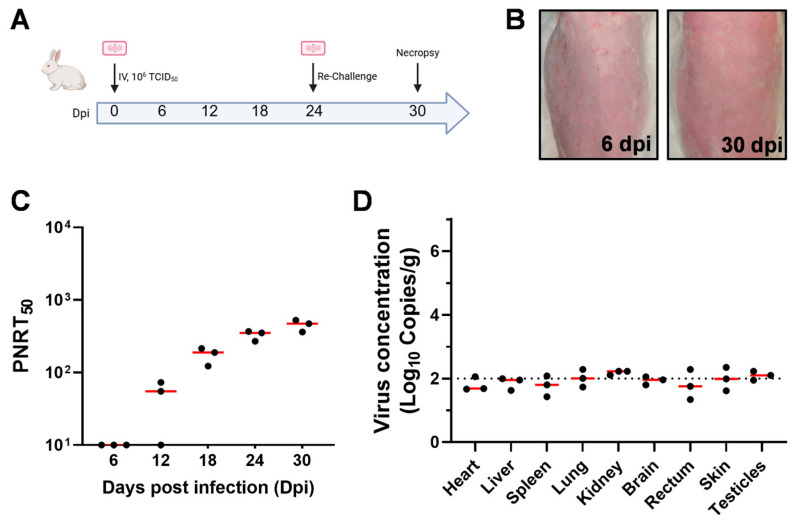
MPXV infection provides protection against reinfection in rabbits. (**A**) Rabbits were infected with 10^6^ TCID_50_ of MPXV via intravenous injection, and then were reinfected in the same manner 24 days after the initial infection. (**B**) Skin lesions of rabbits at 6 dpi and on the 6th day after the reinfection (i.e., 30 dpi). (**C**) The PNRT_50_ neutralizing antibody titers of rabbits at different time points after infection. (**D**) The viral DNA copy numbers in the tissues and organs of rabbits at 30 dpi were determined by q-PCR.

To determine whether rabbits develop protective immunity against reinfection after an initial infection, we administered a high-dose intravenous injection and boosted the infection in the same manner 24 days later (Figure 5A). The results showed that the rabbits exhibited skin lesions at 6 dpi, whereas no lesions were observed on day 6 after reinfection (Figure 5B). The neutralizing antibodies in rabbits were effectively activated after the initial infection and continued to rise during the follow-up period; reinfection further boosted these neutralizing antibody levels (Figure 5C). Moreover, we did not detect viral load in the various organs (Figure 5D), suggesting that the adaptive immunity activated after the initial infection effectively protected them from reinfection with MPXV. This result indicates that the rabbit model has the potential for evaluating MPXV vaccines.

## 4. Discussion

Previous studies have demonstrated that the intravenous injection of MPXV in adult rabbits effectively induces symptoms such as fever and rash, mimicking the self-limiting course of human mpox [13]. However, gaps remain in understanding how the peripheral immune response is initiated and the corresponding molecular change characteristics in rabbits during MPXV infection [13]. Herein, we investigated the transcriptomic changes in rabbit PBMCs upon MPXV infection and analyzed the conserved molecular signatures across different MPXV infection models.

In the transcriptomic analysis of rabbits at 6 dpi with MPXV, pathways related to pathogenic infection and inflammatory response were enriched, and inflammatory genes such as *IL1A*, *IL1B*, and *CXCL8* were significantly upregulated. At 14 dpi, terms including “T cell receptor signaling pathway” were enriched. In the rhesus macaque infection model, interferon and inflammatory response pathways in PBMCs were activated within the first 7 days, while terms associated with T cell activation were enriched at 7 dpi and 14 dpi [12]. These pieces of evidence reflect the transition of PBMCs from innate immunity to adaptive immunity post-infection. Previous studies have reported that monocytes exhibit low susceptibility to MPXV [14]. In lethal MPXV-infected cynomolgus monkey models, peripheral monocytes and granulocytes are the primary poxvirus-positive cells, whereas PBMCs from surviving cynomolgus monkeys are virus-negative [8]. In the non-lethal rabbit model, PBMCs also showed resistance to viral infection, suggesting that the observed transcriptomic changes are likely due to the immune response rather than the direct viral infection of PBMCs. Whether the infection of monocytes within PBMCs contributes to the fatal outcome of mpox requires further investigation.

By comparing the PBMC transcriptomes of rabbits and rhesus macaques, this study identified, for the first time, the conserved molecular signatures of these two species during the mpox acute phase. Interferons have been shown to reduce MPXV pathogenicity in CAST/EiJ mice and rhesus macaques [10]. IFN pathway-related genes, such as ISG15 and members of the OAS and IFIT families, were upregulated in PBMCs of both infected animal species. This cross-species conservation highlights their core role in MPXV clearance. Downregulated DEGs included the *MS4A1* and *FCRL* families, which are involved in B cell activation and antibody production. Currently, no studies have reported the dynamic changes in B cells in the early stage of mpox. These genes could serve as diagnostic markers for mpox. Additionally, rabbits developed protective immunity against reinfection 24 days after primary MPXV infection, indicating that this model can be used for mpox vaccine evaluation.

This study has limitations: the small numbers of animals per group as well as the short follow-up time. There may also be important differences between experimental MPXV infection in rabbits and humans. The cellular resolution of RNA sequencing is limited, failing to distinguish the molecular expression patterns of specific cell subsets. Further validation of the conserved molecules in human samples is required.

In conclusion, this study provides insights into the molecular characteristics of PBMC changes in the MPXV-infected rabbit model, highlights the response pattern of PBMCs transitioning from innate to adaptive immunity, identifies cross-species conserved molecular signatures, and validates the value of the rabbit model for mpox vaccine evaluation. These findings offer references for mpox diagnosis, vaccine development, and therapeutic research.

## Data Availability

All data generated and analyzed in this study are included in this article.
